# IFN-λ4 potently blocks IFN-α signalling by ISG15 and USP18 in hepatitis C virus infection

**DOI:** 10.1038/s41598-017-04186-7

**Published:** 2017-06-19

**Authors:** Pil Soo Sung, Seon-Hui Hong, Jae-Hee Chung, Sojeong Kim, Su-Hyung Park, Ho Min Kim, Seung Kew Yoon, Eui-Cheol Shin

**Affiliations:** 1 Graduate School of Medical Science and Engineering, KAIST, Daejeon, 34141 Republic of Korea; 2 Division of Hepatology, Department of Internal Medicine, Seoul St. Mary’s Hospital, The Catholic University of Korea, Seoul, 06591 Republic of Korea; 3 BioMedical Science and Engineering Interdisciplinary Program, KAIST, Daejeon, 34141 Republic of Korea; 4 Department of Biological Sciences, KAIST, Daejeon, 34141 Republic of Korea

## Abstract

Genetic polymorphisms in *IFNL4* have been shown to predict responses to IFN-α-based therapy in hepatitis C virus (HCV)-infected patients. The *IFNL4*-ΔG genotype, which encodes functional IFN-λ4 protein, is associated with a poor treatment response. In the present study, we investigated the induction and biological effects of IFN-λ4 in HCV-infected hepatocytes and their association with responsiveness to IFN-α. We also studied the effects of direct-acting antiviral (DAA) treatment on IFN-λ4 expression and IFN-α responsiveness. HCV infection induced IFN-λ4 expression at mRNA and protein levels in primary human hepatocytes (PHHs). In hepatoma cells, *IFNL4* gene transfection or recombinant IFN-λ4 protein treatment robustly increased the protein levels of ISG15 and USP18 in an IFNLR1-dependent manner and potently blocked IFN-α signalling. The ISG15/USP18-mediated IFN-α unresponsiveness was demonstrated by transfection of siRNAs targeting ISG15 and/or USP18. This potent IFN-λ4 effect was related to prolonged ISG expression after *IFNL4* gene transfection. DAA treatment of HCV-infected PHHs reduced the expression of IFN-λs, including IFN-λ4, and restored IFN-α responsiveness. These results demonstrate that virus-induced IFN-λ4 potently blocks IFN-α signalling by inducing high protein levels of ISG15 and USP18. Moreover, the data clearly demonstrate that DAA therapy restores IFN-α responsiveness in HCV-infected cells.

## Introduction

The global prevalence of hepatitis C virus (HCV) infection is approximately 3%^1^. The majority of patients with HCV infection fail to clear the virus and develop chronic persistent infections^2^. Until recently, the standard treatment for HCV infection was a combination of pegylated interferon-α (peg-IFN-α) and ribavirin. Currently, HCV infection is usually treated with various direct-acting antivirals (DAAs) that target different HCV proteins, and a high rate of sustained virological response (SVR) is achieved with these drugs^3^. Unfortunately, the high cost of DAAs has resulted in limited access in developing countries where the disease burden is relatively high^4^. DAAs can also lead to the emergence of HCV resistance-associated variants (RAVs). DAA failure usually occurs in less than 5% of treated chronic hepatitis C patients, and RAVs are found in most of these cases^5, 6^.

After HCV infects hepatocytes, the expression of interferons (IFNs) and IFN-stimulated genes (ISGs) is induced despite the interference mechanisms of the virus^2^. Several studies have demonstrated that in cell culture models, HCV infection induces the production of IFNs, with IFN-λs expressed at higher levels than IFN-β^7–10^. IFN-λs are produced as long as HCV persists in the host, and the infected liver has high levels of many ISGs^2^.

Notably, 50% of patients with genotype 1 HCV infection fail to achieve a SVR with peg-IFN-α-based therapy. In 2009, it was found that single nucleotide polymorphisms (SNPs) located near the *IFNL3* locus are strongly linked with the response to peg-IFN-α-based therapy^11–13^. However, the mechanisms by which the SNPs near the *IFNL3* locus influenced treatment outcome were unknown until the *IFNL4* gene was identified near the *IFNL3* locus in 2013^14^. The expression of the IFN-λ4 protein, which is encoded by the *IFNL4* gene, is influenced by a germline dinucleotide frameshift variant located in exon 1 of the *IFNL4* gene (*rs368234815*)^14^. The *rs368234815*-∆G allele generates the full-length IFN-λ4 protein, whereas the *rs368234815*-TT allele does not generate the IFN-λ4 protein because of a premature stop codon^14^. A recent study showed that *IFNL4*-ΔG/TT is the *IFNL* gene polymorphism that is primarily responsible for the treatment response to peg-IFN-α-based therapy^15^. Chronic hepatitis C patients with the *rs368234815*-∆G allele (∆G/TT or ∆G/∆G), which encodes the functional IFN-λ4 protein, respond poorly to peg-IFN-α-based therapy^14, 15^.

*In vitro* experiments using the recombinant IFN-λ4 protein showed that IFN-λ4 activates the Janus kinase (JAK)-signal transducer and activator of transcription (STAT) signalling pathway by binding to the IFN-λ receptor^16^ and induces the expression of ISGs^17^. As expected, the hepatic levels of ISGs in HCV-infected livers are associated with functional IFN-λ4 expression^18^, and a functionally impaired variant of IFN-λ4 is associated with weaker induction of ISGs in HCV-infected livers^19^. These genotype-phenotype correlation studies demonstrate that functional IFN-λ4 protein is the driver of high hepatic ISG expression as well as the cause of poor treatment response. However, there have been no mechanistic studies that could explain why the *rs368234815*-∆G allele encoding the IFN-λ4 protein is associated with a poor response to peg-IFN-α-based treatment.

The present study investigated the expression of IFN-λ4 in HCV-infected hepatocytes and the relationship of the biological effects of IFN-λ4 with IFN-α responsiveness. We also studied the effects of DAA treatment on the expression of IFN-λ4 and IFN-α responsiveness in HCV-infected hepatocytes.

## Results

### HCV infection results in IFN-λ4 expression and IFN-α unresponsiveness

**Figure 1:**
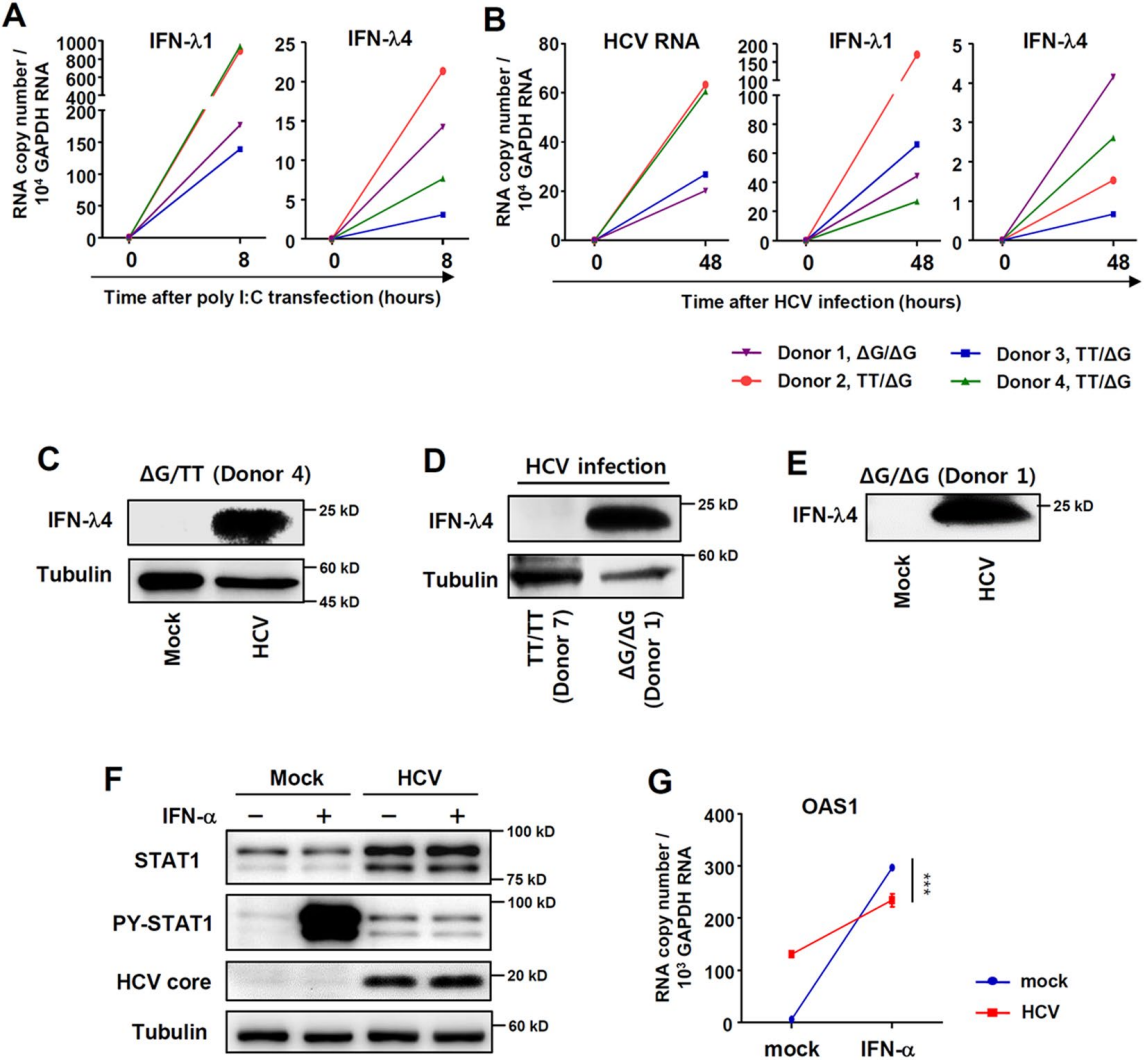
HCV infection results in IFN-λ4 expression and IFN-α unresponsiveness. (**A**) PHHs from 4 different donors were transfected with poly(I:C) (6 µg/ml). After 8 hours, the cells were harvested, and gene expression was analysed by real-time qPCR. (**B**) PHHs from 4 different donors were infected with JFH1 HCVcc at 10 MOI. After 48 hours, the cells were harvested, and gene expression was analysed by real-time qPCR. (**C**) PHHs with *IFNL4* ΔG/TT genotype were infected with JFH1 HCVcc at 10 MOI. After 72 hours, the cell lysate was harvested, and protein expression were analysed by immunoblotting. (**D**) PHHs from two different donors (one with *IFNL4* TT/TT genotype and the other with *IFNL4* ΔG/ΔG genotype) were infected with JFH1 HCVcc at 10 MOI. After 72 hours, the cell lysates were harvested, and protein expression were analysed by immunoblotting. (**E**) PHHs with *IFNL4* ΔG/TT genotype were infected with JFH1 HCVcc at 10 MOI. After 72 hours, the culture supernatant was harvested, and IFN-λ4 protein expression was analysed by immunoblotting. The data are representative of two independent experiments. (**F**,**G**) PHHs with the *IFNL4* ΔG/ΔG genotype were infected with JFH1 HCVcc at 5 MOI. After 72 hours, the cells were treated with 100 IU/ml IFN-α for 30 minutes (**F**) or 6 hours (**G**). The cells were harvested, and gene and protein expression were analysed by immunoblotting (**F**) or real-time qPCR (**G**). Data are presented as means ± S.E.M. ***P ≤ 0.001 (Student’s t-test). All the data are representative of two independent experiments. Full-length blots of PY-STAT1 (**F**) with multiple exposure times are included in the Supplementary Fig. 9A.

First, we examined the expression of type I and type III IFNs after HCVcc infection in primary human hepatocytes (PHHs). In line with the previous studies^7–10^, we confirmed the induction of IFN-λs, rather than IFN-β (Supplementary Fig. 1). Next, using PHHs from four different donors with *IFNL4* ΔG/ΔG or TT/ΔG genotypes, we observed that both poly(I:C) transfection (Fig. 1A) and cell culture-derived HCV (HCVcc) infection (Fig. 1B) induced IFN-λ4 mRNA expression, although the mRNA level of IFN-λ4 was much lower than that of IFN-λ1 (Fig. 1A,B). We also examined time kinetics of IFN-λ4 gene expression after HCVcc infection (Supplementary Fig. 2). We confirmed the expression of IFN-λ4 after HCVcc infection at the protein level in PHHs with *IFNL4* ΔG allele (Fig. 1C,D) whereas PHHs with *IFNL4* TT/TT genotype did not produce IFN-λ4 protein after HCVcc infection (Fig. 1D). Furthermore, we detected IFN-λ4 protein in culture supernatant of PHHs with *IFNL4* ΔG/ΔG genotype after HCVcc infection (Fig. 1E). We reported previously that prolonged stimulation with IFN-λ3 blocks the response to exogenous IFN-α^10^. To investigate whether the endogenous IFN-λ proteins that are produced in response to HCV infection, including the IFN-λ4 protein, also render cells nonresponsive to exogenous IFN-α, we infected PHHs with HCVcc and examined the response to exogenous IFN-α. Both STAT1 phosphorylation (Fig. 1F) and OAS1 upregulation (Fig. 1G) in response to exogenous IFN-α were attenuated in HCV-infected PHHs.

### DAA treatment restores IFN-α unresponsiveness caused by HCV infection

**Figure 2:**
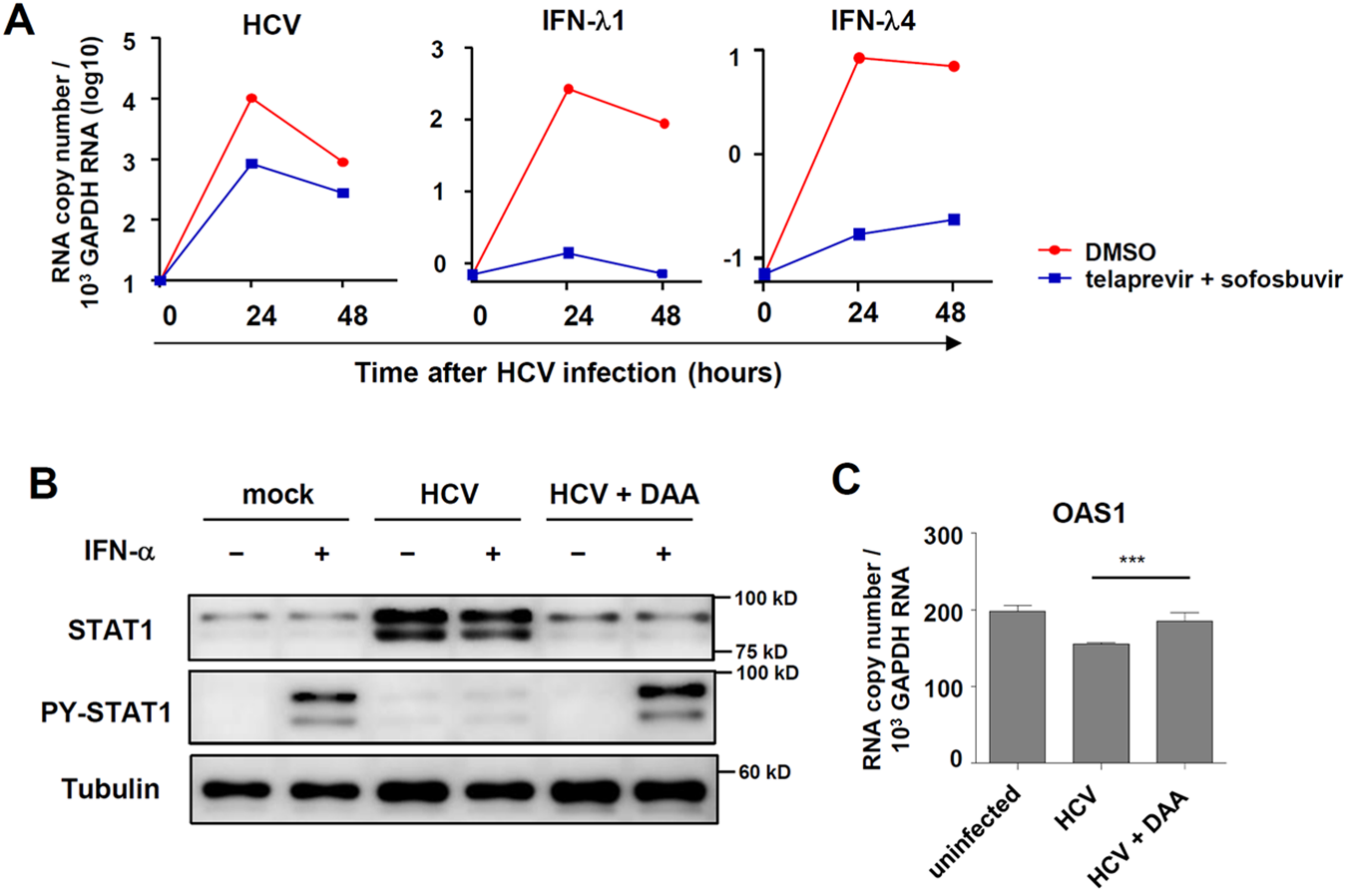
HCV infection results in IFN-λ4 expression and IFN-α unresponsiveness that is restored by DAA treatment. (**A**) PHHs were infected with JFH1 HCVcc at 2 MOI. Shortly after HCV infection, telaprevir (5 μM) plus sofosbuvir (5 μM) were added to HCV-infected cells. After 24 or 48 hours, the cells were harvested, and gene expression was analysed by real-time qPCR. (**B**) PHHs with the *IFNL4* ΔG/ΔG genotype were infected with JFH1 HCVcc at 2 MOI. Six hours after HCV infection, the culture medium was changed to the medium containing telaprevir (5 μM) and sofosbuvir (5 μM). After 120 hours, the cells were treated with 100 IU/ml of IFN-α for 30 minutes (**B**) or 6 hours (**C**). The cells were harvested, and gene and protein expression were analysed by immunoblotting (**B**) or real-time qPCR (**C**). Data are presented as means ± S.E.M. ***P ≤ 0.001 (Student’s t-test). DAA: direct-acting antivirals. All the data are representative of two independent experiments. Full-length blots of PY-STAT1 (**B**) with multiple exposure times are included in the Supplementary Fig. 9B. The data are representative of three independent experiments.

Next, we examined the effect of DAAs on IFN-λ4 expression in HCV-infected PHHs. Treatment with telaprevir and sofosbuvir reduced the intracellular HCV RNA titre, resulting in greatly attenuated IFN-λ1 and IFN-λ4 gene expression (Fig. 2A). These data indicate that viral replication is critical for the induction of IFN-λ expression in HCV-infected PHHs. Moreover, treatment with telaprevir and sofosbuvir restored IFN-α responsiveness, as demonstrated by restored STAT1 phosphorylation (Fig. 2B) and OAS1 upregulation (Fig. 2C). Taken together, these data demonstrate that DAA treatment reduces the expression of IFN-λs and restores IFN-α responsiveness in HCV-infected cells.

### IFN-λ4 induces ISG expression

**Figure 3:**
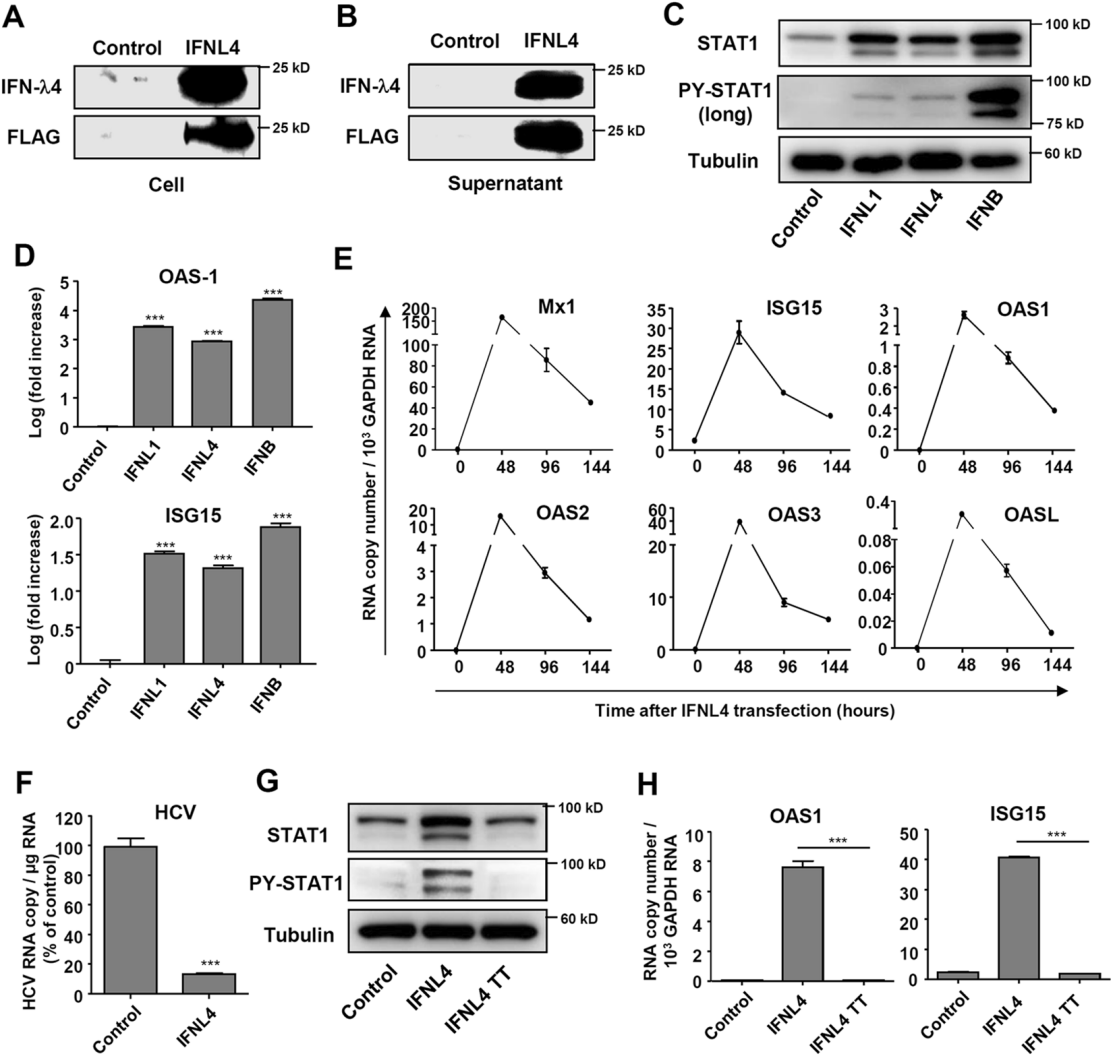
IFN-λ4 overexpression induces ISG expression. (**A**,**B**) Huh-7 cells were transfected with control or IFN-λ4-expressing plasmids. After 72 hours, the cell lysates (**A**) and culture supernatants (**B**) were harvested, and protein expression were analysed by immunoblotting. For the culture supernatants, immunoprecipitation with anti-FLAG-conjugated beads were used to pull down the FLAG-tagged IFN-λ4 protein. The data are representative of two independent experiments. (**C**,**D**) Huh-7 cells were transfected with control, IFN-λ1-, IFN-λ4-, or IFN-β-expressing plasmids. After 96 hours, the cells were harvested, and protein expression or gene expression were analysed by immunoblotting (**C**) or real-time qPCR (**D**). (**E**) Huh-7 cells were transfected with IFN-λ4-expressing plasmids. The cells were harvested at the indicated time points, and gene expression was analysed by real-time qPCR. (**F**) Huh-7 cells were infected with JFH1 HCVcc at 0.5 MOI followed by transfection with control or IFN-λ4-expressing plasmids. After 72 hours, the cells were harvested, and HCV RNA titer was analysed by real-time qPCR. (**G**,**H**) Huh-7 cells were transfected with control, IFN-λ4-, or IFN-λ4 TT-encoding plasmids. After 96 hours, the cells were harvested, and protein and gene expression were analysed by immunoblotting (**G**) or real-time qPCR (**H**). In (**D**,**E**,**F**,**H**) data are presented as means ± S.E.M. ***P ≤ 0.001 (Student’s t-test). All the data are representative of two independent experiments. Full-length blots of PY-STAT1 (**C**) with multiple exposure times are included in the Supplementary Fig. 9C.

To examine the effect of IFN-λ4, we first transfected the IFN-λ4-expressing plasmid into Huh-7 hepatoma cells of which *IFNL4* genotype is TT/TT. We also transfected the cells with IFN-β- or IFN-λ1-expressing plasmids for comparative purposes. As results, we detected IFN-λ4 protein in cell lysates after transfection with the IFN-λ4-expressing plasmid (Fig. 3A). Moreover, we detected IFN-λ4 protein in culture supernatant by immunoprecipitation and immunoblotting (Fig. 3B) although IFN-λ4 protein is known to be poorly secreted when it is expressed in mammalian cells^14, 20, 21^. Transfection with the IFN-λ4-expressing plasmid induced STAT1 phosphorylation (Fig. 3C) and ISG expression (Fig. 3D) in both Huh-7 cells and in PHHs (Supplementary Fig. 3). Our previous report showed that HCV-infected hepatocytes produce IFN-λs that increase the protein levels of STAT1, STAT2, and IRF9, and that increased levels of STAT1, STAT2, and IRF9 prolong the upregulation of some ISGs^10^. Transfection with the IFN-λ4-expressing plasmid also caused sustained upregulation of these ISGs, including Mx1, ISG15, OAS1, OAS2, OAS3, and OASL (Fig. 3E). In fact, the intracellular HCV RNA titre was decreased by transfection with the IFN-λ4-expressing plasmid (Fig. 3F). Furthermore, we used the *IFNL4*-TT plasmid, which cannot generate IFN-λ4 protein due to a premature stop codon^14^ and found that it did not cause STAT1 phosphorylation (Fig. 3G) nor did it induce ISG expression (Fig. 3H). STAT1 phosphorylation and induction of ISG expression were also observed by treatment with recombinant IFN-λ4 protein (Supplementary Fig. 4). Silencing IFNLR1 expression (Supplementary Fig. 5A) markedly decreased ISG expression (Supplementary Fig. 5B) and STAT1 phosphorylation (Supplementary Fig. 5C) in IFN-λ4 gene-transfected cells and STAT1 phosphorylation (Supplementary Fig. 5D) in recombinant IFN-λ4-treated cells, confirming that IFNLR1 is required for the action of IFN-λ4.

### Cells that overexpress IFN-λ4 or treated with IFN-λ4 show weaker responses to IFN-α treatment

**Figure 4:**
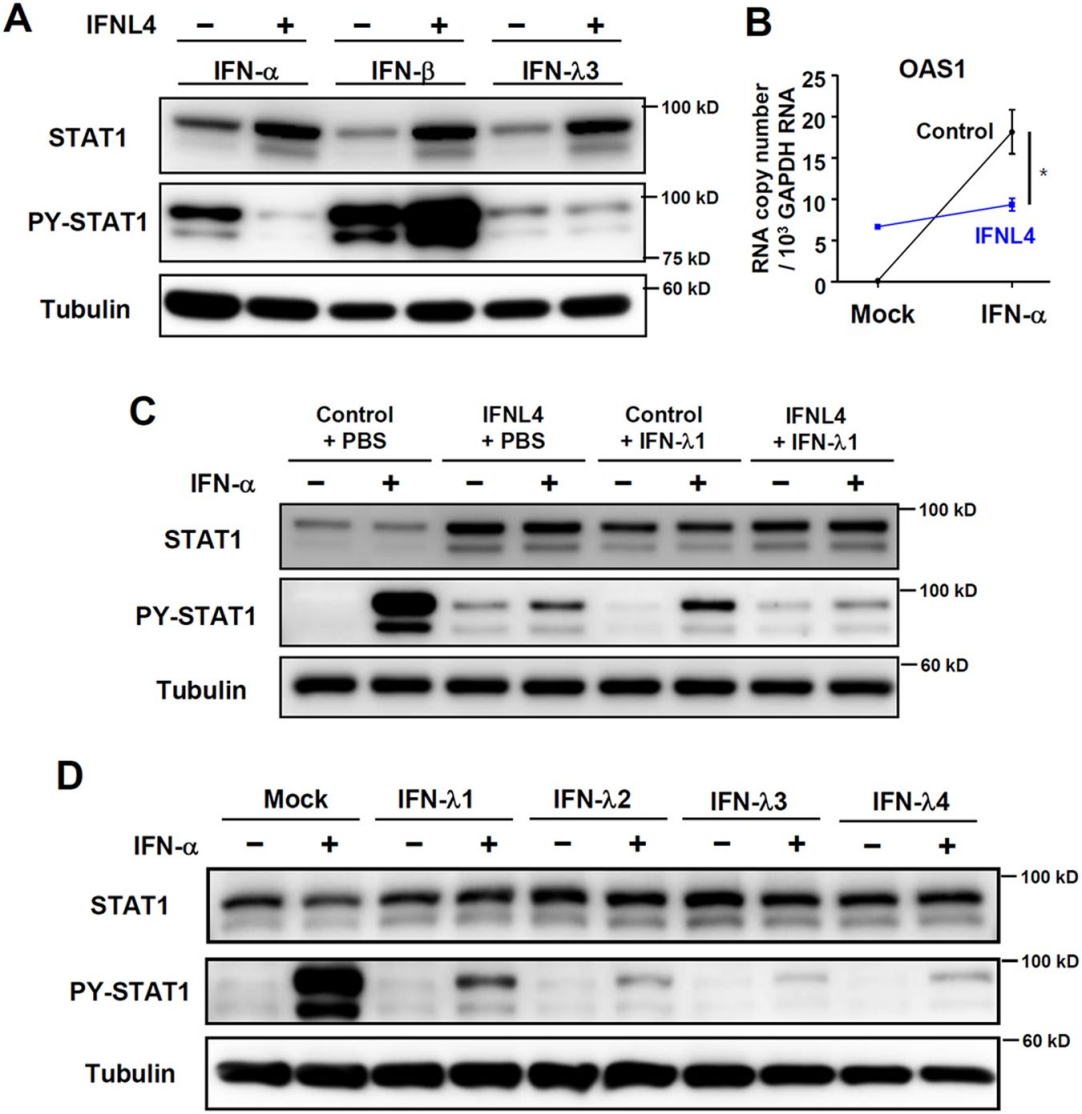
Cells that overexpress IFN-λ4 or treated with IFN-λ4 show weaker responses to IFN-α treatment. (**A**,**B**) Huh-7 cells were transfected with control or IFN-λ4-expressing plasmids. After 96 hours, the cells were treated with 100 IU IFN-α, 1 ng/mL IFN-β, or 100 ng/mL IFN-λ3. The cells were harvested after 15 minutes, and protein expression was analysed by immunoblotting (**A**), or the cells were harvested after 6 hours, and gene expression was analysed by real-time qPCR (**B**). Data are presented as means ± S. E. M. *P ≤ 0.05, ***P ≤ 0.001 (Student’s t-test). (**C**) Huh-7 cells were transfected with control or IFN-λ4-expressing plasmids. After 24 hours, the transfected cells were treated with PBS or IFN-λ1 (10 ng/ml). After 48 hours, the cells were treated with 100 IU IFN-α for 15 minutes. The cells were harvested, and protein expression was analysed by immunoblotting. All the data are representative of two independent experiments. (**D**) Huh-7 cells were treated with mock or 10 ng/mL recombinant IFN-λ1, 2, 3, or 4. After 72 hours, the cells were treated with 100 IU IFN-α. The cells were harvested after 30 minutes, and protein expression was analysed by immunoblotting. All the data are representative of two independent experiments.

We reported previously that IFN-λ3-pretreated cells show an attenuated response to exogenous IFN-α treatment, as evidenced by reduced STAT1 phosphorylation and ISG expression compared to non-pretreated cells^10^. Similarly, IFN-λ4-transfected cells also showed weaker STAT1 phosphorylation (Fig. 4A), weaker STAT2 phosphorylation (Supplementary Fig. 6A) and lower ISG expression (Fig. 4B) in response to exogenous IFN-α treatment compared to mock-transfected control cells. However, this unresponsiveness was shown only in response to IFN-α treatment, not to IFN-β or -λ treatment (Fig. 4A). IFN-λ4-transfected cells were also unresponsive to peg-IFN-α-2b treatment (Supplementary Fig. 6B). IFN-λ1 pre-treatment also resulted in an attenuated response to exogenous IFN-α, and the response to IFN-α was almost completely blocked by the combination of IFN-λ1 pre-treatment and IFN-λ4 gene transfection (Fig. 4C). This indicates the importance of additional IFN-λ4 protein in *IFNL4*-∆G/TT or *IFNL4*-∆G/∆G hosts. We also confirmed the unresponsiveness to exogenous IFN-α by IFN-λs using recombinant IFN-λ proteins including IFN-λ4 (Fig. 4D).

### IFN-α unresponsiveness depends on ISG15 and USP18 in cells that overexpress IFN-λ4 or treated with IFN-λ4

**Figure 5:**
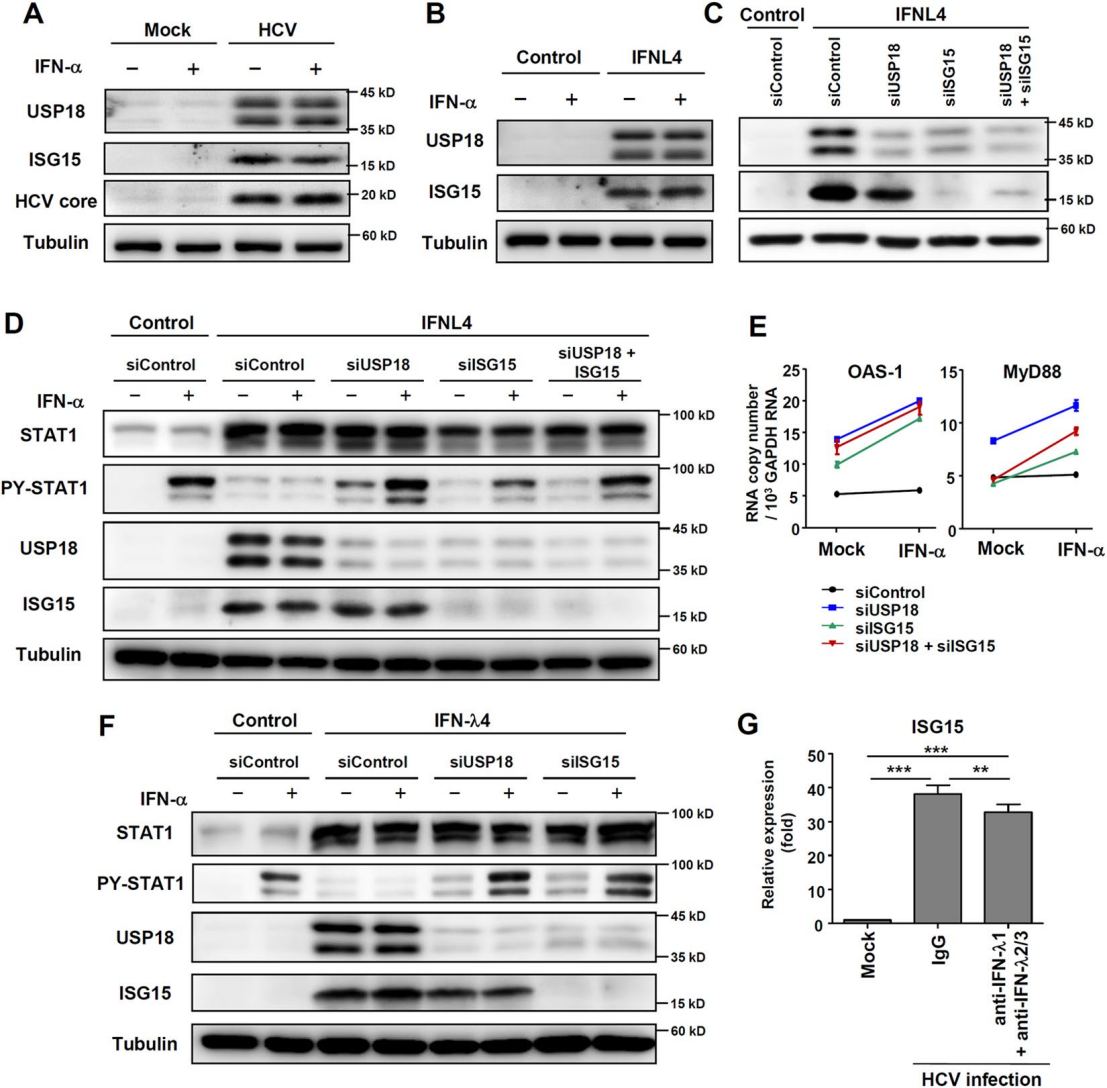
IFN-α unresponsiveness depends on ISG15 and USP18 in cells that overexpress IFN-λ4 or treated with IFN-λ4. (**A**) PHHs with the *IFNL4* ΔG/ΔG genotype were infected with JFH1 HCVcc at 5 MOI. After 72 hours, the cells were treated with 100 IU/ml of IFN-α for 30 minutes. (**B**) Huh-7 cells were transfected with control or IFN-λ4-expressing plasmids. After 72 hours, the cells were treated with 100 IU IFN-α for 15 minutes. The cells were harvested, and protein expression was analysed by immunoblotting. (**C**) Huh-7 cells were transfected with control, USP18-, or ISG15-targeting siRNAs. After 24 hours, the cells were transfected with control or IFN-λ4-expressing plasmids. After 48 hours, the cells were re-transfected with the respective siRNAs. The cells were harvested, and gene expression was analysed by immunoblotting 24 hours after the second siRNA transfection. (**D**,**E**) Huh-7 cells were transfected with control, USP18-, or ISG15-targeting siRNAs. After 24 hours, the cells were transfected with control or IFN-λ4-expressing plasmids. After 48 hours, the cells were re-transfected with the respective siRNAs. After 48 hours, the cells were treated with 100 IU/ml of IFN-α for 15 minutes (**D**) or for 6 hours (**E**). Then the cells were harvested, and protein and gene expression were analysed by immunoblotting (**D**) or real-time qPCR (**E**). All the data are representative of two independent experiments. (**F**) Huh-7 cells were treated with or without 100 ng/mL recombinant IFN-λ4. After 48 hours, the cells were transfected with control siRNAs, USP18-, or ISG15-targeting siRNAs. The USP18-, or ISG15-targeting siRNAs used in this experiment had different sequences from siRNAs used in the experiment for (**D**). After 48 hours, the cells were treated with 100 IU/ml of IFN-α for 15 minutes. Then the cells were harvested, and protein expression were analysed by immunoblotting. (**G**) PHHs with IFNL4 ΔG/ΔG genotype were infected with JFH1 HCVcc at 15 MOI. After 6 hours, the cells were treated with 10 μg/ml anti-IFN-λ1 and 10 μg/ml anti-IFN-λ2/3 or IgG for 72 hours. The cells were harvested, and ISG15 gene expression was analysed by real-time qPCR. Data are presented as means ± S.E.M. **P ≤ 0.01, ***P ≤ 0.001 (Student’s t-test).

We recently observed that prolonged IFN-λ3 stimulation induces robust and sustained upregulation of ISG15, which stabilizes the USP18 protein and diminishes the response to exogenous IFN-α treatment^10^. Here we found that the USP18 and ISG15 protein levels were upregulated in HCV-infected PHHs (Fig. 5A) and in IFN-λ4-transfected cells (Fig. 5B), which was expected in light of the previous observations. We next performed experiments to confirm the roles of ISG15 and USP18 in IFN-λ4-induced IFN-α unresponsiveness. As expected from the previous study^10^, silencing ISG15 expression decreased the protein level of USP18 in IFN-λ4-transfected cells (Fig. 5C). Silencing the expression of ISG15 or USP18 expression restored STAT1 phosphorylation (Fig. 5D) and ISGs expression (Fig. 5E; Supplementary Fig. 7A) upon exogenous IFN-α treatment. However, silencing other ISGs, such as mitochondrial antiviral-signaling protein (MAVS) or interferon regulatory factor 3 (IRF3), did not restore exogenous IFN-α-induced ISG expression (Supplementary Fig. 7B). The response to exogenous IFN-α was more strongly restored by silencing USP18 and ISG15 expression at the same time (Fig. 5D,E). We confirmed these results in recombinant IFN-λ4-treated cells using different siRNAs targeting USP18 or ISG15 (Fig. 5F).

We also confirmed that ISG15 maintains USP18 protein levels independently of its ISGylating activity. ISG15 silencing decreased the amount of USP18 protein in recombinant IFN-λ4-treated cells, and the protein level of USP18 was restored not only by transfection of wild type (WT) ISG15 gene but also by transfection of conjugation-defective ISG15 AA mutant gene (Supplementary Fig. 8A). As a result, WT ISG15- or AA mutant-transfected cells showed refractoriness against exogenous IFN-α treatment (Supplementary Fig. 8A). Furthermore, we confirmed that USP18 protein interferes with IFN-α signaling irrespective of its enzymatic activity. USP18 silencing restored phosphorylation of STAT1 after IFN-α treatment, and forced expression of WT USP18 or enzymatically inactive C64S USP18 mutant blocked phosphorylation of STAT1 (Supplementary Fig. 8B).

Finally, we examined relative importance of IFN-λ4 among the members of the IFN-λ family in the induction of ISG15 during HCV infection using a mixture of anti-IFN-λ1 and anti-IFN-λ2/3 which neutralize the effect of IFN-λ1, -λ2, and -λ3. As a result, ISG15 expression was highly maintained even after addition of a mixture of neutralizing anti-IFN-λ1 and anti-IFN-λ2/3 in HCV-infected cells. This result indicates that ISG15 upregulation is derived and maintained by IFN-λ4 produced during HCV infection.

## Discussion

In this study, we show that HCV infection induces IFN-λ4 expression and that IFN-λ4 potently attenuates the response to exogenous IFN-α treatment by the action of ISG15 and USP18. DAA treatment of HCV-infected cells reduced the expression of IFN-λs, including IFN-λ4, and restored IFN-α responsiveness.

We and other groups have reported robust production of IFN-λs, rather than IFN-β, at both the mRNA and protein levels in HCV-infected PHHs^7–10^. It was demonstrated previously that IFN-λ4 expression is induced by poly(I:C) transfection into PHHs^14, 21^, but it has not been known whether IFN-λ4 expression is induced by HCV infection in PHHs. We have demonstrated for the first time that IFN-λ4 expression is induced by HCV infection in PHHs.

A recent study reported that *IFNL4*-ΔG/TT is the primary *IFNL4* gene polymorphism determining the treatment response to peg-IFN-α-based therapy^15^. In HCV-infected patients, the *IFNL4*-TT genotype, which does not produce IFN-λ4 protein, is associated with a favourable response to IFN-α-based therapy. The *IFNL4*-ΔG genotype, which encodes functional IFN-λ4 protein, is associated with a poor treatment response. However, the mechanisms underlying this paradoxical association remained unclear. Our data explain why the presence of functional IFN-λ4 protein in *IFNL4*-ΔG genotype-carrying hosts is associated with a poor response to IFN-α-based HCV treatment. Moreover, our data suggest that DAA therapy can restore IFN-α responsiveness in HCV-infected patients.

HCV-infected patients with *IFNL4*-∆G allele exhibit high baseline levels of ISGs in their livers^2^. Among the proteins enriched in HCV-infected liver, USP18 has been reported to confer unresponsiveness to IFN-α by interacting with IFNAR2 and inhibits downstream signals induced by IFN-α^22^. In fact, USP18 overexpression decreases the responsiveness to exogenous IFN-α^23^. Recent reports show that intracellular free ISG15 protein maintains the stability of the USP18 protein in human cells^24, 25^, although ISG15 has direct antiviral properties against many viruses in mice^26^. ISG15 deficiency caused increased viral resistance in humans but not mice, since only human ISG15 interacted with USP18 and decreased the response to IFN-α^25^. More specifically, in human cells, free ISG15 stabilizes the USP18 protein by preventing its ubiquitination, leading to an attenuated response to IFN-α^24, 25^. Importantly, the ISGylation activity of ISG15 and the deISGylation activity of USP18 do not have any role in this process. It was shown that ISGylation-defective ΔGG ISG15 also binds to USP18 and prevents its degradation^24^. Furthermore, previous studies reported that blocking of IFN-α signaling by USP18 does not depend on the enzymatic activity of USP18^27^. The IFN-α signal-blocking activity of USP18 is mediated by the binding of USP18 to the intracellular domain of IFNAR2, which prevents the binding of JAK1 to IFNAR2^27^.

Our group recently found that prolonged stimulation of IFN-λ3 induces the robust and sustained upregulation of ISG15 that stabilizes the USP18 protein and causes the unresponsiveness to exogenous IFN-α treatment^10^. We also reported that both ISG15 and USP18 protein levels are increased in HCV-infected livers^10^. In the current study, for the first time, we demonstrated that ISG15 and USP18 protein levels are increased in HCV-infected PHHs (Fig. 5A) and IFN-λ4-expressing (Fig. 5B) or -treated cells (Fig. 5F) and that ISG15 and USP18 proteins mediate IFN-α unresponsiveness in IFN-λ4-expressing or -treated cells (Fig. 5D–F). Recently, Fan *et al*. also demonstrated that IFN-λ4 inhibits the JAK–STAT signalling pathway by inducing USP18 expression^28^. However, our current study showed the expression of IFN-λ4 after HCV infection for the first time. In addition, we observed IFN-α unresponsiveness in a more natural system for HCV infection. We infected PHHs with HCVcc and observed IFN-α unresponsiveness which was caused by endogenously produced IFNs, including IFN-λ4. Moreover, we identified ISG15 as a critical regulator for increased USP18 protein levels in IFN-λ4-expressing or -treated cells and demonstrated for the first time that DAAs can restore IFN-α responsiveness by decreasing the production of endogenous IFN-λs, including IFN-λ4, in HCV-infected cells.

Taken together, our data clearly demonstrate that virus-induced IFN-λ4 potently blocked IFN-α signalling via upregulation of ISG15 and USP18 in HCV infection and that DAA therapy restored the IFN-α responsiveness of HCV-infected cells. These results explain why HCV-infected patients with the *IFNL4 rs368234815*-∆G allele encoding the IFN-λ4 protein respond poorly to peg-IFN-α-based treatment. In the era of DAAs, the use of oral, IFN-free DAA regimens has become the standard of care in treating HCV infection, and IFN-α-based regimens are no longer the first-line treatment option. However, the current data suggest that an IFN-α-based regimen might still be beneficial in combination with DAAs or as rescue treatment of DAA-failed patients.

## Materials and Methods

### Cell lines, primary human hepatocytes, and reagents

Huh-7 cells (Apath), HepG2 cells, and A549 cells were maintained at 37 °C with 5% CO_2_ in Dulbecco’s modified Eagle medium (DMEM) containing 10% foetal bovine serum (WelGENE), 4.5 g/l glucose, L-glutamine, and 1% penicillin/streptomycin (WelGENE). Frozen vials of PHHs from 7 different donors were purchased from Invitrogen. The lot numbers and the *IFNL4* genotypes of the PHHs are shown in Supplementary Table 1. After thawing, the PHHs were centrifuged at 100× g for 10 minutes and incubated in collagen-coated 24-well plates overnight. The PHHs were maintained in Williams E Medium containing hepatocyte maintenance supplement reagents (Invitrogen). Small-interfering RNAs (siRNAs) against IFNLR1, USP18, ISG15, and scrambled sequences were obtained from Santa Cruz Biotechnology. Poly(I:C) was purchased from InvivoGen. All transfections were performed using the jetPRIME transfection reagent (Polyplus transfection) via a reverse transfection method for Huh-7 cells and a forward transfection method for PHHs. The siRNA transfections were performed using lipofectamine RNAi MAX (Invitrogen). Recombinant IFN-α-2a was obtained from PBL Assay Science, recombinant IFN-β was obtained from PeproTech, peg-IFN-α-2b was obtained from MSD, and recombinant human IFN-λ1, -λ2, and -λ3 were obtained from R&D Systems. Recombinant human IFN-λ4 was produced from Expi293F cells with glycosylation modification. Plasmids encoding IFN-β, IFN-λ1, and IFN-λ4 were synthesized by GenScript. IL-28A, IL-29 ELISA kits were purchased from RayBiotech (Norcross, GA), and IFN-β ELISA kit was purchased from Fujirebio (Tokyo, Japan).

### Production and infection of HCVcc

The Japanese fulminant hepatits-1 (JFH-1) strain (genotype 2a) of HCVcc was produced as described previously^10^. DMEM containing 5% human serum was used to culture the Huh-7.5 cells to produce highly infectious JFH1 HCVcc. HCVcc infectivity was quantified by a colorimetric focus-forming assay as described previously^29^. Huh-7 cells were infected with JFH-1 HCVcc at 0.5 multiplicity of infection (MOI), and PHHs were infected at 2–10 MOI.

### Immunoblotting

To prepare total cell lysates, the cells were lysed with RIPA buffer (Thermo Fisher Scientific). Ten micrograms of each cell lysate were loaded on SDS-PAGE gels. The antibodies used for immunoblotting were as follows: IFN-λ4 (1:200, mouse, Millipore MABF227), IFN-λ4 (1:200, rabbit, Abcam ab196984), FLAG (1:500, mouse, Sigma, F3165), STAT1 (1:1000, rabbit, BD Biosciences 610120), PY-STAT1 (1:1000, mouse, BD Biosciences 612233), STAT2 (1:1000, rabbit, Santa Cruz Biotechnology sc-476), IRF9 (1:1000, rabbit, Santa Cruz sc-496), ISG15 (1:1000, mouse, Santa Cruz Biotechnology sc-69701), USP18 (1:1000, rabbit, Cell Signaling 4813S), HCV core (1:1000, mouse, Thermo MA1-080), tubulin (mouse, Sigma T6074), horseradish peroxidase (HRP)-conjugated rabbit IgG (1:5000, Abcam ab97051), HRP-conjugated mouse IgG (1:5000, Abcam ab97023). For immunoprecipitation of FLAG-tagged IFN-λ4 protein, anti-FLAG M2 Affinity Gel (Sigma) was used.

### RNA extraction, cDNA synthesis, and real-time quantitative PCR

Total RNA isolation, cDNA synthesis, and TaqMan real-time quantitative PCR were performed as described previously^30^. In brief, total RNA was isolated with the GeneAll Ribospin^TM^ (GeneAll), and cDNA was synthesized using a cDNA synthesis Master kit (LeGene). TaqMan Gene Expression Assays (Applied Biosystems) were used to determine the mRNA levels of target genes. Quantification of intracellular HCV RNA copies was performed as described previously^30^. The results were standardized to the mRNA levels of GAPDH, and the data are presented as means ± standard error of the mean (s.e.m.). The quantification of mRNA copies of IFN-λs was performed as described previously^18^. We used PCR primers and TaqMan probe that were validated to detect only IFN-λ4 (p179) with IFNL4-ΔG/ΔG genotype or p123 in TT/TT genotype^18^ as follows: forward primer CGA TCC TGG AGC TGC TG; reverse primer TTT GTG ACG CCT CTT CTGG; TaqMan probe: GAG GGA TGT GGC GGC CT.

### Statistical analyses

Data from experiments with cell lines and PHHs are presented as means ± s.e.m. Unpaired t-tests or two-tailed Mann-Whitney U-tests were performed for statistical analysis. All of the analyses were performed using GraphPad Prism version 5.01. *P* values less than 0.05 were considered to be statistically significant.

## Electronic supplementary material

Supplementary Information
