# Supplementary material for: IFN-λ4 potently blocks IFN-α signalling by ISG15 and USP18 in hepatitis C virus infection
Source: Sci Rep. 2017 Jun 19;7:3821. doi: 10.1038/s41598-017-04186-7 (PMC5476576; doi:10.1038/s41598-017-04186-7)
Supplement: Supplementary file 1 — Supplementary Information [file 41598_2017_4186_MOESM1_ESM.pdf]

SREP-16-45737 Supplementary Information

IFN-λ4 potently blocks IFN-α signalling by ISG15 and USP18 in hepatitis C virus infection

Pil Soo Sung[1, 2, †], Seon-Hui Hong[3, †], Jae-Hee Chung[4], Sojeong Kim[3], Su-Hyung Park[1], Ho Min Kim[1], Seung Kew Yoon[2], Eui-Cheol Shin[1, 3,*]

[1]Graduate School of Medical Science and Engineering, KAIST, Daejeon 34141, Republic of Korea

[2]Division of Hepatology, Department of Internal Medicine, Seoul St. Mary's Hospital, The Catholic University of Korea, Seoul 06591, Republic of Korea

[3]BioMedical Science and Engineering Interdisciplinary Program, KAIST, Daejeon 34141, Republic of Korea

[4]Department of Biological Sciences, KAIST, Daejeon 34141, Republic of Korea

[†]These authors contributed equally to this work.

Supplementary Figure 1

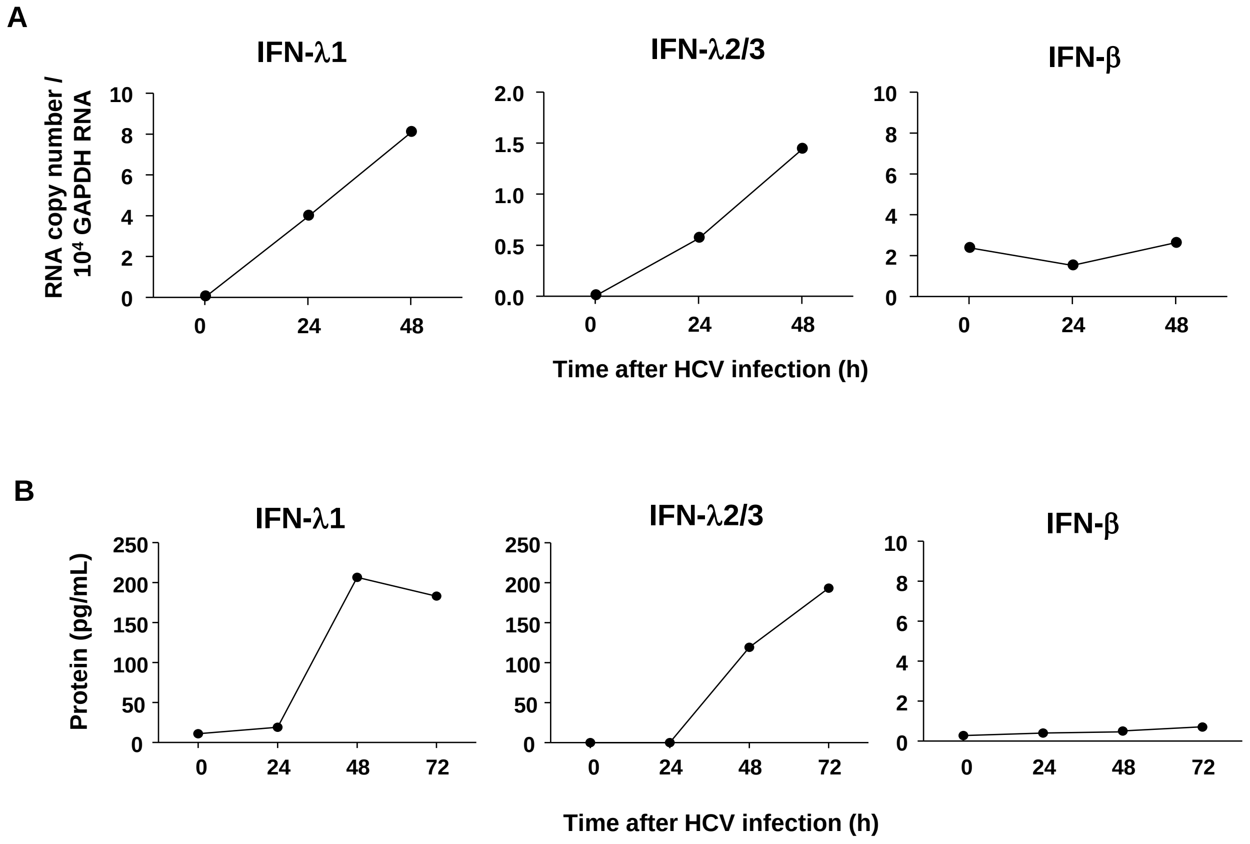

Supplementary Figure 1. Expression of type I and type III IFNs in HCV-infected PHHs.

PHHs were infected with JFH1 HCVcc at 15 MOI. Cell pellets and culture supernatants were harvested at indicated time points. Gene expression was analysed by real-time qPCR, and secreted protein was analysed by ELISA.

Supplementary Figure 2

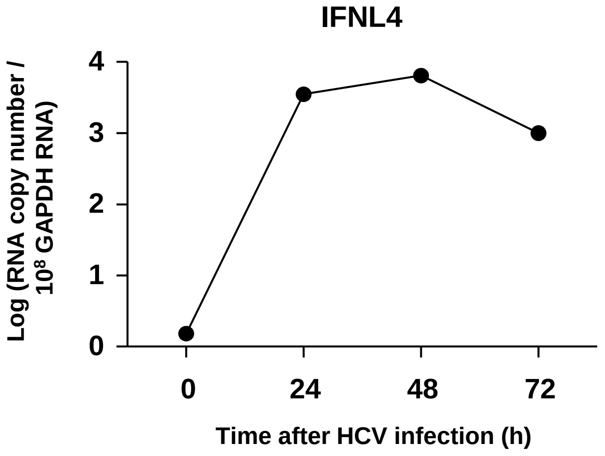

Supplementary Figure 2. Time kinetics of IFNL4 gene expression in HCV-infected PHHs.

PHHs were infected with JFH1 HCVcc at 10 MOI. The cells were harvested, and IFNL4 gene expression was analysed by real-time qPCR. Data are presented as means ± S. E. M.

Supplementary Figure 3

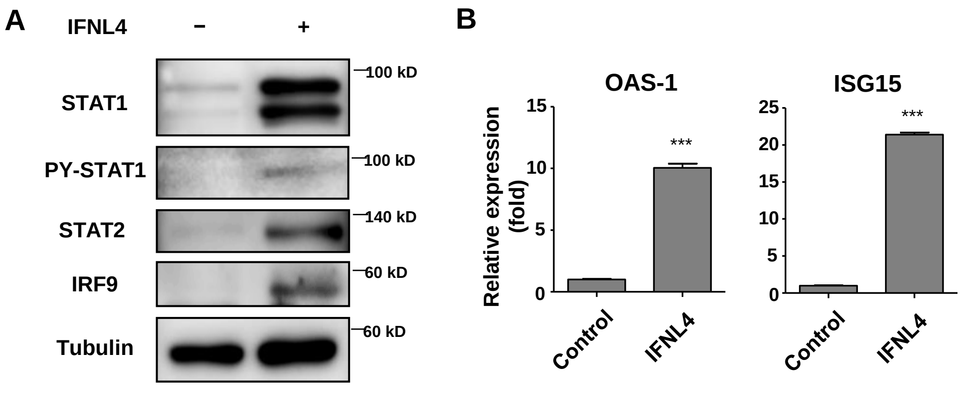

Supplementary Figure 3. IFN-λ4 overexpression in PHHs induces ISG expression. (A, B) PHHs were transfected with control or IFN-λ4-expressing plasmids. After 72 hours, the cells were harvested, and protein and gene expression were analysed by immunoblotting (A) or real time-qPCR (B). Data are presented as means ± S. E. M. ***$P \leq 0.001$ (Student's t-test).

Supplementary Figure 4

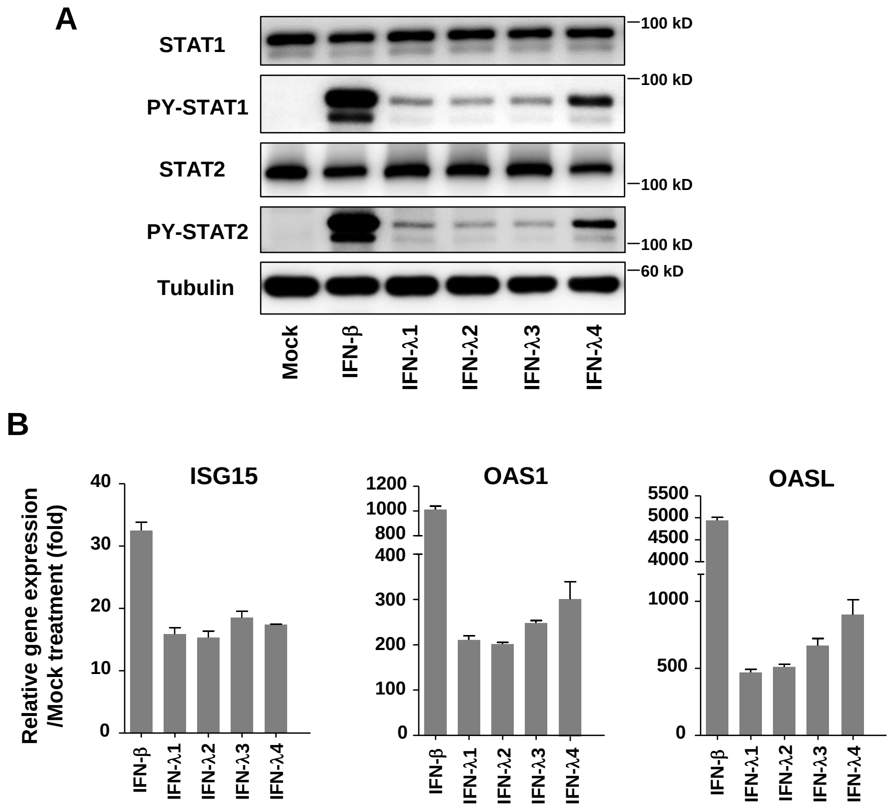

Supplementary Figure 4. Recombinant IFN-λ4 activates STAT phosphorylation and induces ISGs.

(A) Huh-7 cells were treated with 3 ng/mL of recombinant IFN-β , or 100 ng/mL of recombinant human IFN-λ1, -λ2, -λ3, or -λ4 protein for 20 minutes (A) or 6 hours (B). The cells were harvested, and protein expression was analysed by immunoblotting (A), and mRNA expression was analysed by real-time qPCR (B). In (B), data are presented as means ± S. E. M.

Supplementary Figure 5

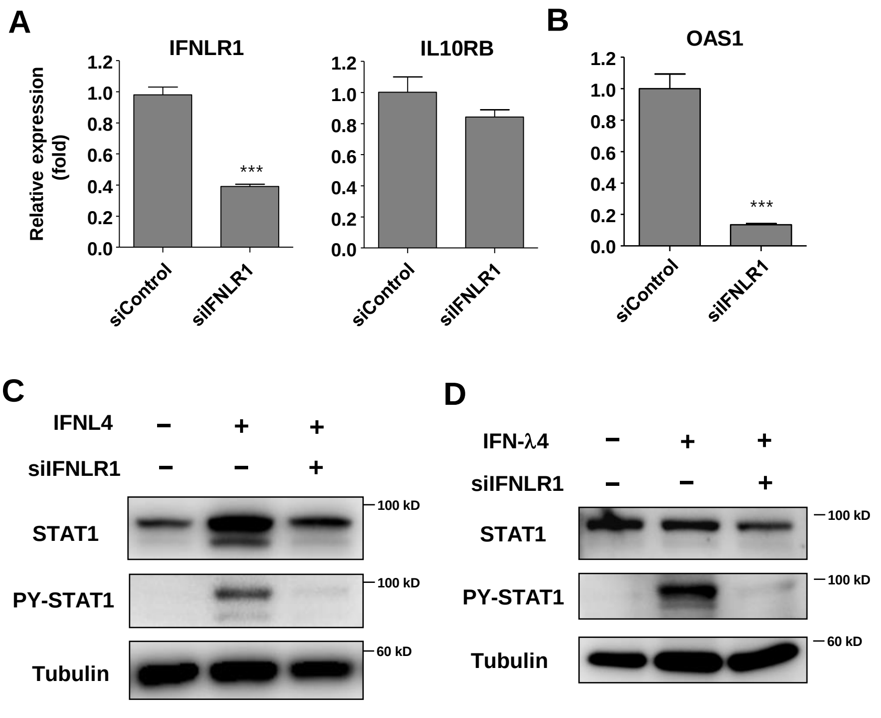

Supplementary Figure 5. IFN-λ receptor is required for the action of IFN-λ4.
(A, B, C) Huh-7 cells were transfected with control or IFNLR1 siRNA. After 24 hours, the cells were transfected with control or IFN-λ4-expressing plasmids. After 72 hours, the cells were harvested, and gene and protein expression were analysed by real-time qPCR (A, B) or immunoblotting (C). In (A) and (B), data are presented as means ± S. E. M. ***$P \leq 0.001$ (Student's t-test). (D) Huh-7 cells were transfected with control or IFNLR1 siRNA. After 48 hours, the cells were treated with 100 ng/mL recombinant human IFN-λ4 protein for 30 minutes. The cells were harvested, and protein expression was analysed by immunoblotting.

Supplementary Figure 6

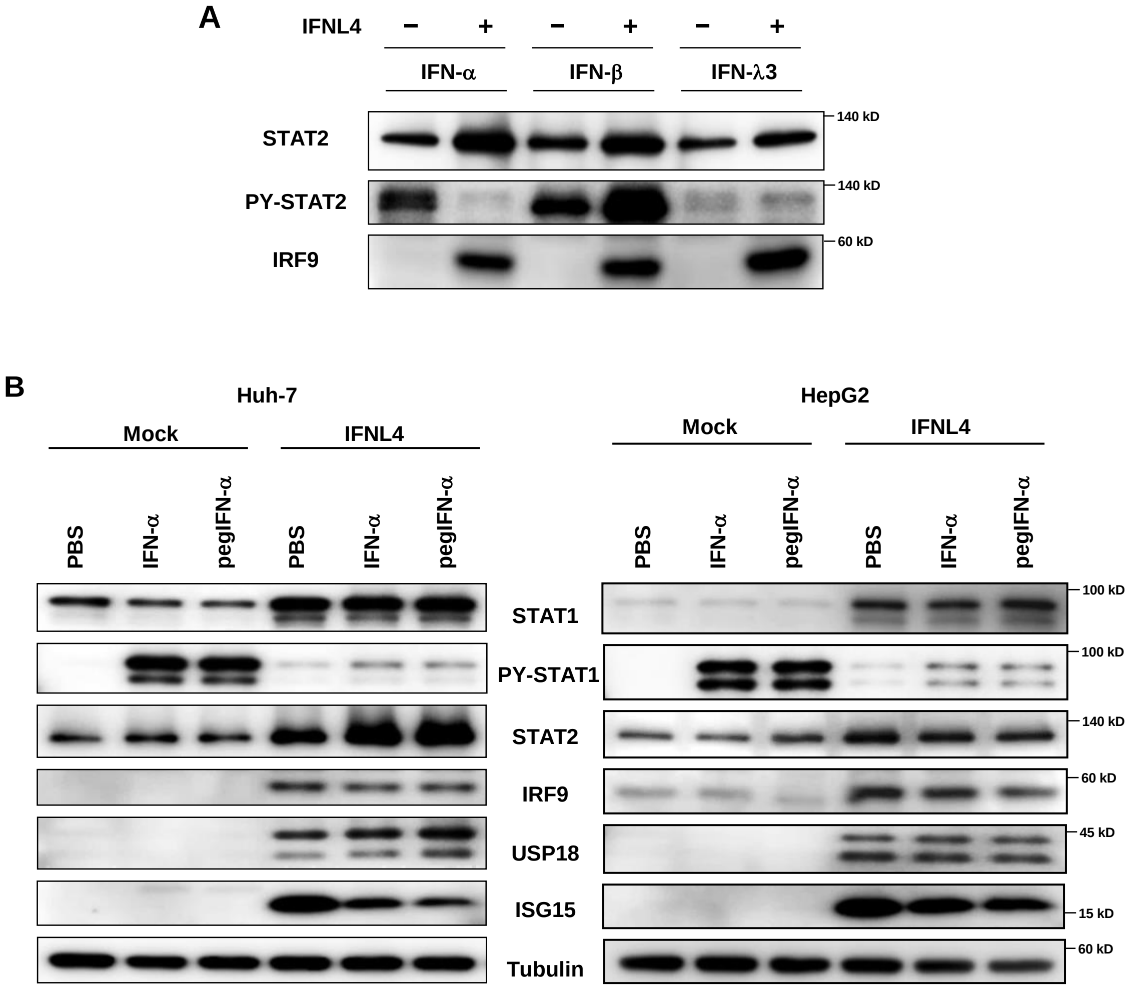

Supplementary Figure 6. Cells that overexpress IFN-λ4 are unresponsive to IFN-α or peg-IFN-α.

(A) Huh-7 cells were transfected with control or IFN-λ4-expressing plasmids. After 96 hours, the cells were treated with 100 IU IFN-α, 1 ng/mL IFN-β, or 100 ng/mL IFN-λ3. The cells were harvested after 15 minutes, and protein expression was analysed by immunoblotting.

(B) Huh-7 cells or HepG2 cells were transfected with control or IFN-λ4-expressing plasmids. After 96 hours, the cells were treated with 100 IU IFN-α or peg-IFN-α. After 15 minutes, the cells were harvested, and protein expression was analysed by immunoblotting.

Supplementary Figure 7

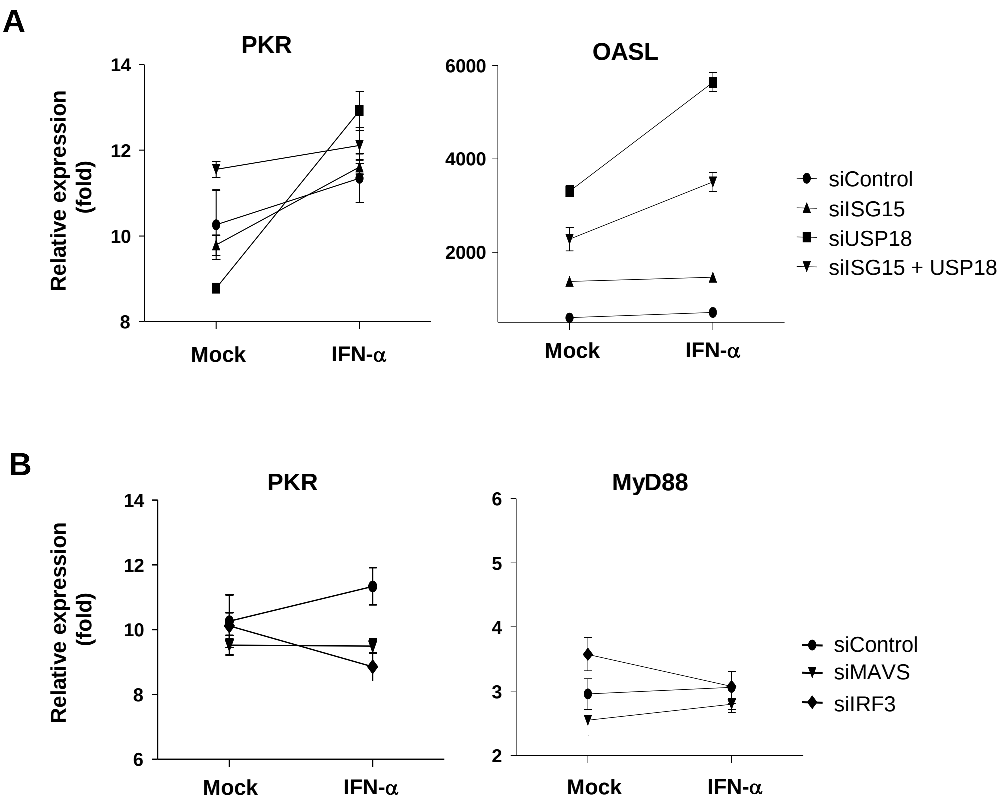

Supplementary Figure 7. IFN-α unresponsiveness depends on ISG15 and USP18 in cells that overexpress IFN-λ4

(A, B) Huh-7 cells were transfected with control, USP18-, or ISG15-targeting siRNAs (A) and control, MAVS-, or IRF3-targeting siRNAs (B). After 24 hours, the cells were transfected with control or IFN-λ4-expressing plasmids. After 48 hours, the cells were re-transfected with the respective siRNAs. After 48 hours, the cells were treated with 100 IU/ml of IFN-α for 6 hours. Then the cells were harvested, and gene expression was analysed by real-time qPCR**.** Data are presented as means ± S.E.M.

Supplementary Figure 8

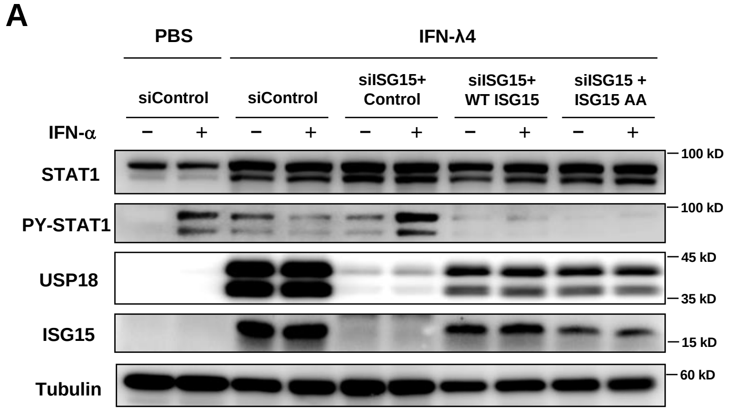

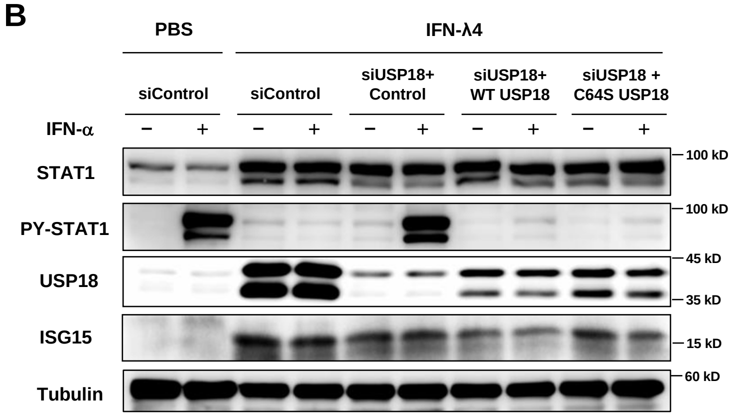

Supplementary Figure 8. ISG15 maintains USP18 independently of its ISGylating activity, and USP18 blocks IFN-α signaling irrespective of its enzymatic activity.
(A) Huh-7 cells were treated with or without 100 ng/mL recombinant IFN-λ4. After 48 hours, the cells were transfected with control siRNAs or ISG15-targeting siRNAs. After 24 hours, the cells were transfected with control plasmid, wild-type (WT) ISG15 plasmid, or conjugation-defective ISG15 AA mutant plasmid. After 48 hours, the cells were treated with 100 IU/ml of IFN-α for 15 minutes. Then the cells were harvested, and protein expression were analysed by immunoblotting. The data are representative of two independent experiments.
(B) Huh-7 cells were treated with or without 100 ng/mL recombinant IFN-λ4. After 48 hours, the cells were transfected with control siRNAs or USP18-targeting siRNAs. After 24 hours, the cells were transfected with control plasmid, WT USP18 plasmid, or enzymatically inactive C64S USP18 mutant plasmid. After 48 hours, the cells were treated with 100 IU/ml of IFN-α for 15 minutes. Then the cells were harvested, and protein expression were analysed by immunoblotting. The data are representative of two independent experiments.

Supplementary Figure 9

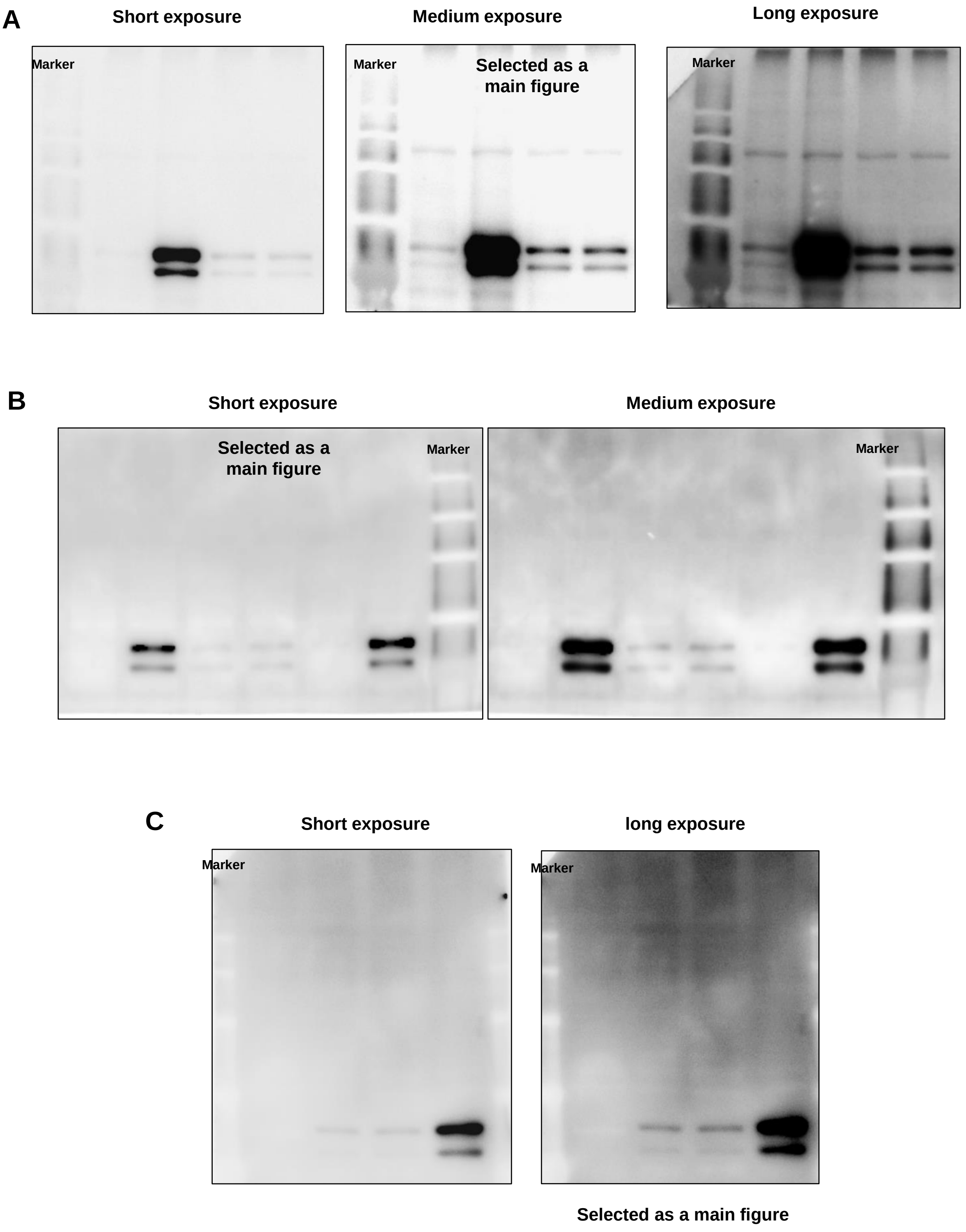

Supplementary Figure 9. Full-length blots of PY-STAT1 with multiple exposure times

Supplementary Table 1

PHH	Lot	*IFNL4* genotype
Donor 1	Hu8200	ΔG/ΔG
Donor 2	Hu1758	TT/ΔG
Donor 3	Hu8196	TT/ΔG
Donor 4	Hu8119	TT/ΔG
Donor 5	Hu8418	TT/TT
Donor 6	Hu8210	ΔG/ΔG
Donor 7	Hu1423	TT/TT

Supplementary Table 1. PHHs from different donors used in this study
